# Toxicology evaluation of radiotracer doses of 3'-deoxy-3'-[^18^F]fluorothymidine (^18^F-FLT) for human PET imaging: Laboratory analysis of serial blood samples and comparison to previously investigated therapeutic FLT doses

**DOI:** 10.1186/1471-2385-7-3

**Published:** 2007-07-03

**Authors:** Eric Turcotte, Linda W Wiens, John R Grierson, Lanell M Peterson, Mark H Wener, Hubert Vesselle

**Affiliations:** 1 Department of Radiology, Division of Nuclear Medicine, University of Washington, Seattle, USA; 2 Department of Laboratory Medicine, University of Washington, Seattle, USA

## Abstract

**Background:**

^18^F-FLT is a novel PET radiotracer which has demonstrated a strong potential utility for imaging cellular proliferation in human tumors in vivo. To facilitate future regulatory approval of ^18^F-FLT for clinical use, we wished to demonstrate the safety of radiotracer doses of ^18^F-FLT administered to human subjects, by: 1) performing an evaluation of the toxicity of ^18^F-FLT administered in radiotracer amounts for PET imaging, 2) comparing a radiotracer dose of FLT to clinical trial doses of FLT.

**Methods:**

Twenty patients gave consent to a ^18^F-FLT injection, subsequent PET imaging, and blood draws. For each patient, blood samples were collected at multiple times before and after ^18^F-FLT PET. These samples were assayed for a comprehensive metabolic panel, total bilirubin, complete blood and platelet counts. ^18^F-FLT doses of 2.59 MBq/Kg with a maximal dose of 185 MBq (5 mCi) were used. Blood time-activity curves were generated for each patient from dynamic PET data, providing a measure of the area under the FLT concentration curve for 12 hours (AUC_12_).

**Results:**

No side effects were reported. Only albumin, red blood cell count, hematocrit and hemoglobin showed a statistically significant decrease over time. These changes are attributed to IV hydration during PET imaging and to subsequent blood loss at surgery. The AUC_12 _values estimated from imaging data are not significantly different from those found from serial measures of FLT blood concentrations (*p *= 0.66). The blood samples-derived AUC_12 _values range from 0.232 ng*h/mL to 1.339 ng*h/mL with a mean of 0.802 ± 0.303 ng*h/mL. This corresponds to 0.46% to 2.68% of the lowest and least toxic clinical trial AUC_12 _of 50 ng*h/mL reported by Flexner et al (1994). This single injection also corresponds to a nearly 3,000-fold lower cumulative dose than in Flexner's twice daily trial.

**Conclusion:**

This study shows no evidence of toxicity or complications attributable to ^18^F-FLT injected intravenously.

## Background

3'-Deoxy-3'-[^18^F]fluorothymidine (^18^F-FLT) is a new tracer for positron emission tomography (PET) being evaluated at several centers across the United States and worldwide. The interest generated by FLT as a radiotracer stems from its potential as a proliferation tracer that would accumulate in tumors in proportion to their growth rate.

FLT, a thymidine nucleoside analog, undergoes the same first metabolic step as thymidine when it is 5'-monophosphorylated by the cytosolic enzyme thymidine kinase-1 (TK-1), however, the 3' substitution prevents further incorporation into DNA. TK-1 is an enzyme within the pyrimidine nucleoside salvage pathway and is up-regulated just prior to and during the DNA synthesis phase of the cell cycle [1-6]. Consequently, TK-1 is closely associated with DNA synthesis and is significantly increased in rapidly proliferating cells and therefore is over-expressed in many tumors. These characteristics led to the evaluation of ^18^F-FLT as a tumor-imaging agent for PET. Grierson and Shields first developed ^18^F-FLT as a potential PET imaging agent [7,8]. ^18^F-FLT has since been demonstrated as a proliferation tracer in tumor cell cultures [9,10] as well as in human lung lesions imaged by PET [11-13]. The potential impact of ^18^F-FLT on tumor imaging is two-fold: first, tumor proliferation is a prognostic factor for certain tumor types (e.g. non-small cell lung cancer [14]) and ^18^F-FLT PET imaging could help refine the prognostic evaluation of these tumors; second, a hallmark of tumors having responded to therapy is a loss of the ability for sustained proliferation. Hence, ^18^F-FLT PET imaging may prove useful in assessing tumor response to therapy.

As a proliferation tracer for PET imaging, ^18^F-FLT is generating much interest but its widespread use will first require an evaluation of its safety when administered in tracer quantities. We have previously reported the dosimetry for ^18^F-FLT, providing radiation dose estimates for human use [15]. A study of the toxicity of ^18^F-FLT administered in tracer quantities for PET has not been reported yet.

FLT was initially investigated as a treatment for HIV infection in humans [16]. Two clinical trials of FLT administered in therapeutic doses revealed evidence of hematologic and hepatic toxicity as well as peripheral neuropathy [17]. The lowest regimen tested was 50 ng*h/mL for a 12 hour dosing interval over 112 days and led to only mild neuropathy within 40 days of therapy. The purpose of this study is to perform an evaluation of the toxicity of ^18^F-FLT administered in radiotracer amounts for PET imaging and to compare such a radiotracer dose of ^18^F-FLT to therapeutic doses previously tested in clinical trials.

## Methods

### Patient selection

This evaluation was conducted as part of a University of Washington IRB approved study of ^18^F-FLT PET in non-small cell lung cancer (NSCLC). All patients were referred with a proven or suspected diagnosis of NSCLC to the University of Washington Medical Center or the Veterans Affairs Puget Sound Health Care System between March 2000 and April 2002. Twenty patients (12 men, 8 women) gave written informed consent to the ^18^F-FLT injection, subsequent PET imaging, and blood draws. The age range for males was 45–81 years (mean 66.6 years) and for females was 46–75 years (mean 60 years). The weight range for males was 54–126 Kg (mean 81.6 Kg) and for females was 46–113 Kg (mean 73.4 Kg). All female subjects were post-menopausal.

### Patient preparation

Study participants had no history of abnormal renal, hepatic or hematologic function. No dietary preparation was required from the patient prior to ^18^F-FLT administration. An intravenous (IV) catheter was placed in each of the patient's arms, the first one for ^18^F-FLT injection and the other to collect blood samples. In eight cases an arterial catheter was used for blood sampling. Patients were hydrated with 500 mL of IV normal saline during the course of the PET imaging procedure.

### ^18^F-FLT synthesis

^18^F-FLT was synthesized according to the method developed by Grierson et al. [7,8] with a minimum acceptable specific activity of 0.1 Ci/μmol. Specific activity was determined by measuring UV absorbance at 266 nm correlated with a radioactivity measurement. The specific activity of FLT was governed by the amount of radioactivity used for FLT synthesis and the time elapsed until patient injection. The specific activity at the time of patient injection had an average value (N = 20) of 0.96 Ci/μmol with a range of 0.12 to 1.6 Ci/μmol. The dose of FLT injected was 0.61 to 8.9 μg (for a 0.07 mCi/Kg radiotracer dose). Each ^18^F-FLT dose passed rigorous radiochemical and chemical purity assays and pyrogenicity and sterility testing. All doses administered contained less than 10% (v/v) ethanol (USP) in no more than a 10 mL solution of isotonic saline.

### PET imaging protocol

All PET studies were performed on a General Electric PET Advance Tomograph (General Electric Medical Systems) using methods described previously [11,15]. All ^18^F-FLT doses were calculated based on patient weight (2.59 MBq/Kg (0.07 mCi/Kg)) with a maximal dose of 185 MBq (5 mCi). ^18^F-FLT was administered intravenously over 1 minute using an infusion pump. A 1.5 or a 2-hour long dynamic acquisition was performed as previously described [15]. Dynamic PET acquisition was accompanied by arterial or venous serial blood sampling. For eight patients imaged over 120 minutes, 24 arterial blood samples were collected as follows: every 20 seconds for the first 3 minutes, every 30 seconds for the next minute, every minute for the next 4 minutes, every 2 minutes for the next 2 minutes, every 5 minutes for the next 10 minutes, every 10 minutes for the next 40 minutes, and at 90 and 110 minutes after injection. For the remaining 12 patients, 8–9 serial venous blood samples were collected at the following times: 1, 2, 5, 10, 20, 40, 60, and 90 minutes for a 90-minute acquisition and at an additional 110 minute time point for a 120-minute acquisition.

### Toxicology testing

Potential changes in the hepatic, hematological or renal function of subjects resulting from administration of 18F-FLT were explored with standard blood laboratory assays. These tests were initially performed within a mean of 5.4 days before 18F-FLT injection as well as just after the PET imaging procedure (mean 2 hours 20 minutes post-injection) (Table 1). The clinical Laboratory Medicine facilities at the University of Washington Medical Center or the Veterans Affairs Puget Sound Health Care System performed these analyses. Subsequent laboratory evaluations acquired as part of the patients' routine care were also reviewed and compared to those obtained earlier. These additional laboratory values fall into one of the following three categories: 1) 5–24 hours, 2) 1–7 days, or 3) greater than 1 week after the 18F-FLT study. Therefore, each patient's blood samples were analyzed: before the PET study, immediately after the PET study, and for at least one of the other three categories listed above (Table 1). The following blood assays were performed: 1) sodium, potassium, chloride, and glucose levels, 2) hepatic function assays (aspartate aminotransferase (AST or SGOT), alanine aminotransferase (ALT or SGPT), albumin, alkaline phosphatase (Alk Phos), and total bilirubin); 3) creatinine and blood urea nitrogen (BUN); 4) hematologic evaluation consisting of a complete blood count including red blood cell count (RBC), hematocrit, hemoglobin, white blood cell count (WBC) and platelet count. An interim statistical analysis carried out on the first 17 patient data sets demonstrated a significant lowering trend for the red blood cell count and hematocrit when comparing values measured before 18F-FLT injection to values obtained just at the end of 18F-FLT imaging. Therefore, a measure of haptoglobin level was added for the last three patients to assess for the presence of hemolysis.

In addition, each patient underwent a standard neurological examination performed by a neurologist or an internist before and immediately after 18F-FLT PET imaging. The evaluation included but was not limited to a Folstein mini mental status examination, as well as evaluations of cranial nerve responses, motor strength, hand strength, muscle bulk and tone, sensation to light and touch, deep tendon reflexes, cognitive testing, Romberg testing, and gait testing. Patients' clinical records for the four months following 18F-FLT PET imaging were also reviewed looking for evidence of new neuropathy or neurological deficit.

From the dynamic PET acquisition data, blood time-activity curves were generated for each patient using regions-of-interest of at least 16 pixels (surface 2.89 cm2) placed in the center of the left ventricular chamber of the heart on each of three adjacent imaging planes [15,18]. Each dataset was corrected for image duration, injected dose, 18F decay, and PET scanner efficiency. The area under the curve (AUC) was calculated for each blood time-activity curve (TAC) for the duration of imaging (90 or 120 minutes) and scaled for a standard 5 mCi 18F-FLT injection. With a specific activity greater than 0.1 Ci/μmol, 5 mCi of 18F-FLT represents a maximum dose of 12.2 μg of FLT injected.

Flexner et al. reported FLT dosing regimens in patients as an area under the blood FLT concentration curve over 12 hours (AUC_12_), since their patients were dosed at 12-hour intervals. Hence, in order to compare our data with Flexner's, we performed an exponential polynomial curve fit (TableCurve 2D, SYSTAT Software Inc.) of the TACs allowing subsequent time integration to be carried out to 12 hours. This approach provided a PET imaging-derived AUC12 for each patient blood TAC.

A separate estimation of the AUC_12 _was calculated from the blood samples collected during PET data acquisition. These samples were counted for radioactivity and decay-corrected according to a method previously described [13]. The resulting blood TACs were also scaled for a standard 5 mCi ^18^F-FLT injection. Curve fitting and integration over 12 hours yielded a blood-derived AUC_12 _value for each patient.

**Table 1:** Time table

	***N***	**Minimum**	**Maximum**	**Mean ± SD**
**Pre-FLT**	20	-3 h 43 m	-37 d	-5.4 d ± 8.7 d
**Immediate <5 h**	18	1 h 24 m	4 h 12 m	2 h 20 ± 41 m
**5 – 24 Hours**	18	5 h 37 m	21 h 24 m	9 h 43 ± 5 h 42
**First Week**	18	1.1 d	6.0 d	4.4 d ± 1.1 d
**> 1 week**	19	7.4 d	155.8 d	29.3 d ± 39.8 d

m = minutes, h = hours, d = days

### Statistical analysis

Laboratory test values were first confirmed for normality using the k-s distance test for each type of clinical laboratory result. Subsequently, parametric tests were used consisting of a one-way Anova test for repeat sampling and a Bonferroni post-test. Comparison between the two methods to calculate AUC_12 _was performed using a Wilcoxon signed rank test. The statistical software packages SPSS (Statistical Package for the Social Sciences, SPSS Inc.) and PRISM (Prism v.4, GraphPad Software) were utilized for this purpose.

## Results

### Laboratory assays

**Figure 1:**
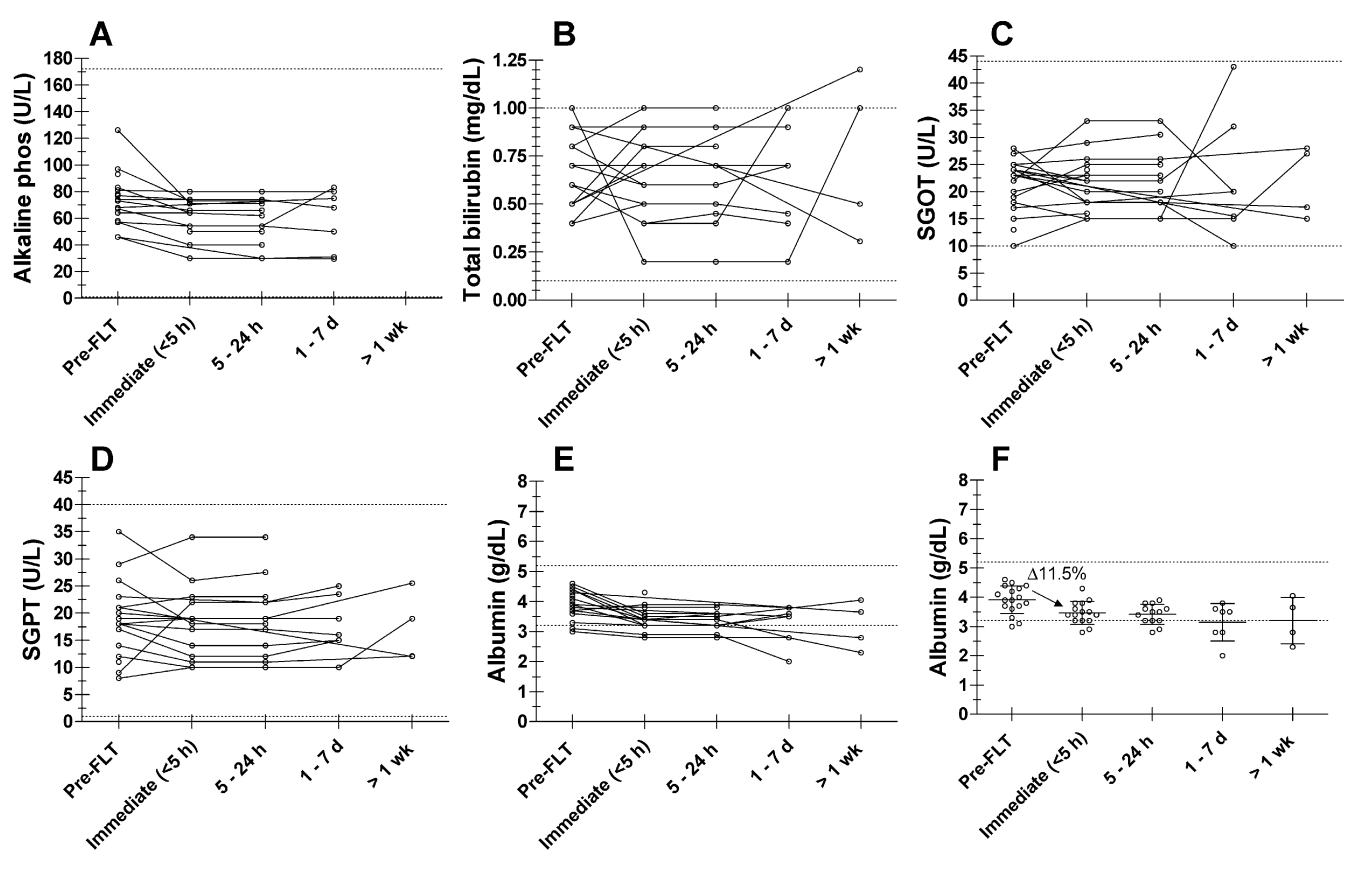
**Hepatic function**. Hepatic function: Alkaline phosphatase (A), total bilirubin (B), SGOT (C), SGPT (D), and albumin (E) levels sampled before FLT injection (Pre-FLT), up to 5 hours post-FLT (Immediate <5 h), between 5 and 24 hours (5–24 h), between day 1 and 7 (1–7 d) and later than one week (>1 wk) after FLT injection. Lines link all values for an individual patient over time. On Figure F (same data as Figure E) the mean +/- standard deviation for albumin is plotted over time. Dotted horizontal lines illustrate the upper and lower normal limits (reference range) for each test.

**Figure 2:**
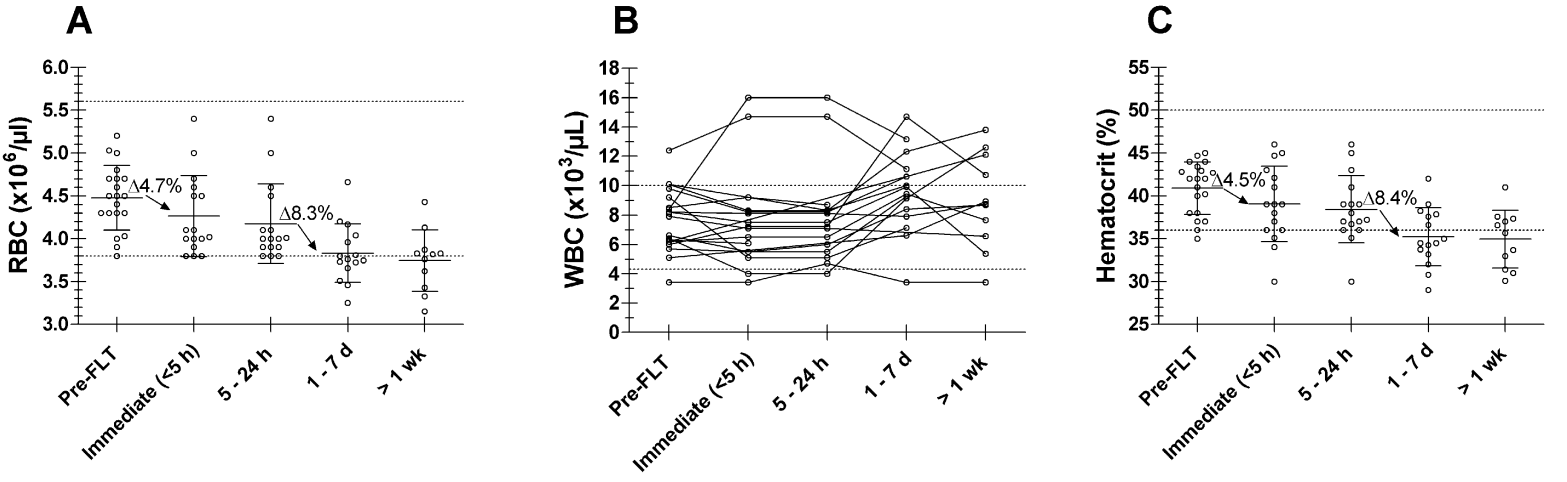
**Hematologic function**. Hematologic function: Red blood cells (A), white blood cells (B), hematocrit (C) levels sampled before FLT injection (Pre-FLT), up to 5 hours post-FLT (Immediate <5 h), between 5 and 24 hours (5–24 h), between day 1 and 7 (1–7 d) and later than one week (>1 wk) after FLT injection. Lines link all values for an individual patient over time. On Figures A & C the means +/- standard deviations for red blood cells (A) and hematocrit (C) are plotted over time. Dotted horizontal lines illustrate the upper and lower normal limits (reference range) for each test.

Different laboratory values monitored for this study are plotted with respect to the time categories for each of the 20 study participants (Figures 1 and 2). Table 2 summarizes the mean and standard deviation for each parameter over time. No statistically significant change was observed in sodium, potassium, chloride, glucose, creatinine, BUN, SGOT, SGPT, Alk Phos, total bilirubin, WBC, and platelet levels (*p *> 0.05). The mean for each of these parameters remained within normal limits over time (Table 2). Albumin, RBC, and hematocrit show a statistically significant decrease over time. Bonferroni analyses demonstrated that albumin decrease results mainly from an initial decrease of 11.5% between the pre-^18^F-FLT PET measure and the immediate post-^18^F-FLT PET blood draw (*p *< 0.001). However, mean albumin values stayed within normal limits. For RBC and hematocrit (as well as for hemoglobin level, not plotted), significant decreases of respectively 4.7% and 4.5% occur between pre-^18^F-FLT and immediate post-^18^F-FLT blood drawn (*p *< 0.001), followed by decreases of respectively 8.3% and 8.4% between 5–24 hours and 1–7 days (*p *< 0.001). However, average levels for RBC remained within normal limits for the 1–7 days time point. Average values were just below normal for RBC after 1 week and for hematocrit for the first week and onwards. Because they are the only laboratory parameters to exhibit a change, raw data, mean and standard deviations (SD) for albumin, RBC, and hematocrit are also plotted for illustration (Figures 1F, 2A, 2C). The effect of surgical intervention on hemoglobin, hematocrit and RBC values was assessed by re-analyzing the data after excluding all post-surgical lab values from the original dataset. Bonferroni post-test performed on this reduced dataset still demonstrates the same significant decrease in hemoglobin, hematocrit and RBC values from pre-^18^F-FLT to immediately post-^18^F-FLT times but without subsequent lowering at later time points. Haptoglobin levels measured for the last 3 patients of the study did not reveal any evidence of hemolysis.

**Table 2:** Laboratory values (mean ± SD) of the 20 patients at each time point and corresponding one-way Anova *P *values across all time points.

	**Pre-FLT**	**Immediate <5 h**	**5 – 24 Hours**	**1 – 7 days**	**> 1 week**	***P***
**Sodium (mEq/L ± SD)**	139.4 ± 1.5	138.2 ± 2.1	138.3 ± 2.0	137.5 ± 1.8	138.1 ± 2.3	0.064
**Potassium (mEq/L ± SD)**	4.2 ± 0.5	4.2 ± 0.4	4.1 ± 0.4	4.2 ± 0.3	4.2 ± 0.4	0.968
**Chloride (mEq/L ± SD)**	102.3 ± 3.3	104.2 ± 3.7	104 ± 3.8	102.3 ± 2.4	101.2 ± 3.1	0.055
**Glucose (mg/dL ± SD)**	95.1 ± 14.8	96.6 ± 20.7	98.5 ± 23.1	105.4 ± 17.7	109.5 ± 14.6	0.175
**Creatinine (mg/dL ± SD)**	0.885 ± 0.198	0.882 ± 0.207	0.881 ± 0.180	0.910 ± 0.190	0.844 ± 0.217	0.949
**BUN (mg/dL ± SD)**	15.8 ± 5.0	15.1 ± 5.6	15.2 ± 6.3	14.3 ± 5.2	15.3 ± 5.7	0.959
**SGOT (U/L ± SD)**	20.8 ± 5.0	22.0 ± 5.1	22.0 ± 5.3	22.2 ± 11.4	21.8 ± 6.7	0.973
**SGPT (U/L ± SD)**	18.7 ± 6.7	18.5 ± 6.6	19.1 ± 6.5	17.6 ± 5.3	17.2 ± 6.5	0.978
**Albumin (g/dL ± SD)**	3.9 ± 0.5	3.5 ± 0.4	3.44 ± 0.3	3.1 ± 0.6	3.2 ± 0.8	0.003
**Alk Phos (U/L ± SD)**	73.8 ± 19.4	61.1 ± 14.7	58.3 ± 17.0	59.5 ± 22.7		0.081
**Bilirubin (mg/dL ± SD)**	0.647 ± 0.181	0.573 ± 0.246	0.581 ± 0.263	0.621 ± 0.286	0.752 ± 0.418	0.714
**RBC (x10**^6^**/μL ± SD)**	4.5 ± 0.4	4.3 ± 0.5	4.2 ± 0.5	3.8 ± 0.3	3.7 ± 0.4	<0.0001
**Hematocrit (% ± SD)**	40.9 ± 3.1	39.1 ± 4.4	38.4 ± 4.0	35.2 ± 3.4	35.0 ± 3.4	<0.0001
**WBC (x10**^3^**/μL ± SD)**	7.6 ± 2.1	7.7 ± 3.4	7.9 ± 3.3	9.5 ± 2.8	9.0 ± 3.2	0.262
**Platelets (x10**^3^**/μL ± SD)**	278.1 ± 96.8	259.1 ± 103.1	255.9 ± 103.0	230.1 ± 76.7	233.5 ± 69.5	0.674

No side effects including nausea, vomiting, dizziness, or headache were reported during ^18^F-FLT injection or during the following 2.0–2.5 hours.

### Neurological evaluations

No change was observed in the results of the neurological examinations performed on all patients before and immediately after the ^18^F-FLT PET imaging sessions. A review of each subject's clinical record covering the 4 months following ^18^F-FLT PET imaging revealed no interval development of new neurological complaints, signs or symptoms by the study participants. In particular, no new peripheral neuropathy was reported in any of the 20 patients.

### Calculation of AUC_12 _for a 5 mCi radiotracer dose of ^18^F-FLT

**Figure 3:**
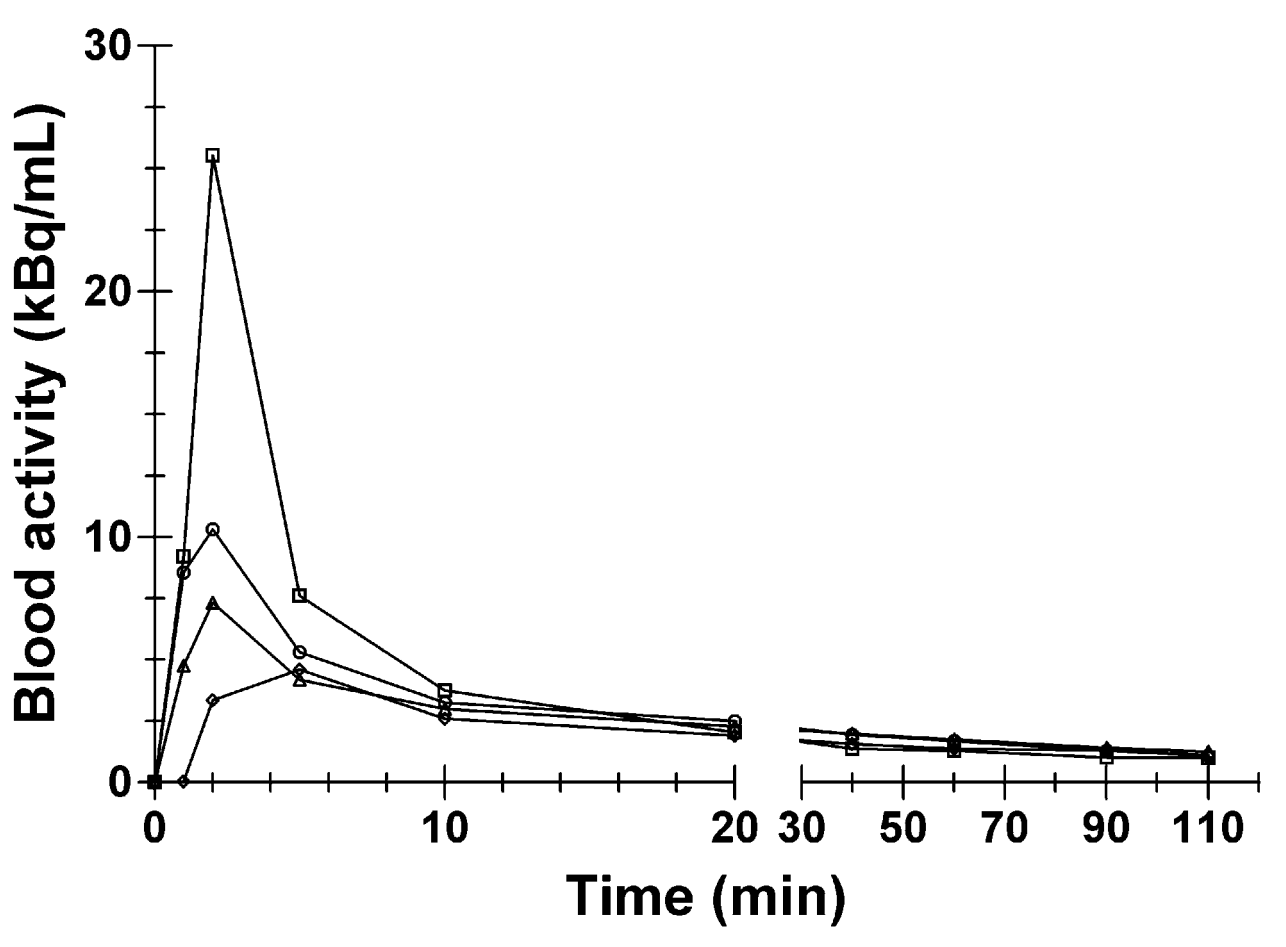
**Examples of TACs**. Blood-derived time-activity curves (TACs) of 4 patients undergoing ^18^F-FLT PET.

Figure 3 depicts 4 blood-derived TACs, each normalized to a standard 5 mCi ^18^F-FLT injected dose. These decay-corrected curves illustrate the variability observed across patients in the elimination of ^18^F-FLT from blood. They also illustrate the observation that at the conclusion of imaging the quantity of residual ^18^F-FLT in the blood was small in all 20 patients. At 90 minutes post-^18^F-FLT injection, the concentration of decay-corrected activity is on average 0.0454 ± 0.0272 μCi/mL. With a lowest acceptable specific activity of 0.1 Ci/μmol, this corresponds to a concentration of FLT of 0.111 ± 0.0665 ng/mL if all radioactivity is assumed to be in the form of ^18^F-FLT (FLT has a molecular weight of 244 g). Therefore, for the purpose of this study we are not considering the main labeled metabolite of FLT, ^18^F-FLT-glucuronide, separately from ^18^F-FLT.

**Figure 4:**
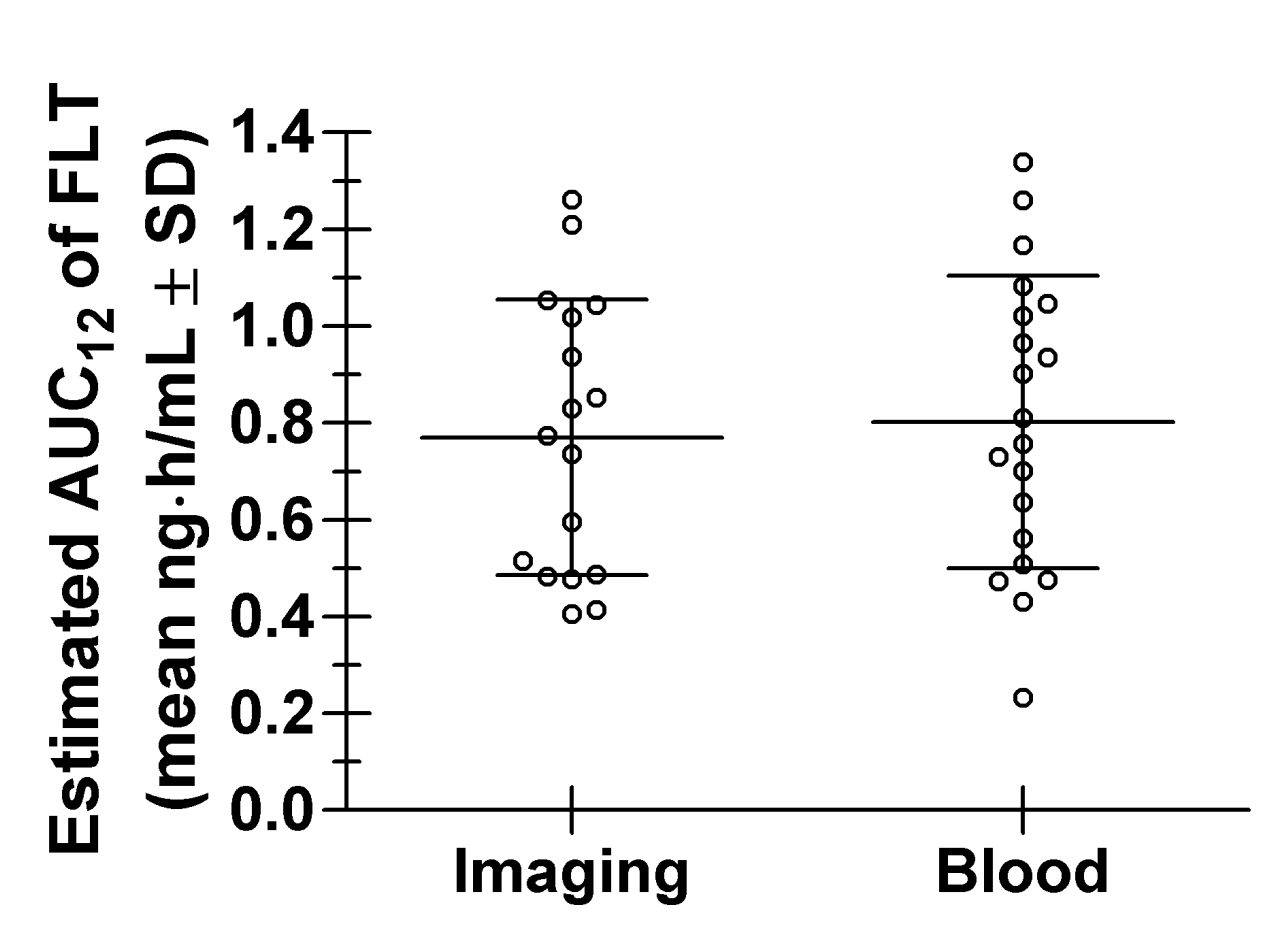
**Comparison of AUC_12 _values derived from imaging with those from blood samples**. Scatter plot comparing image-derived FLT AUC12 (Imaging) values to blood-derived FLT AUC12 (Blood) values for all 20 patients. The means +/- standard deviations are represented by bars.

The imaging-derived AUC_12 _values range from 0.405 ng*h/mL to 1.26 ng*h/mL with a mean of 0.770 ± 0.285 ng*h/mL. The calculated blood sample-derived AUC_12 _ranged from 0.232 ng*h/mL to 1.34 ng*h/mL with a mean of 0.802 ± 0.303 ng*h/mL. No significant difference in the AUC_12 _values obtained by the 2 different methods was found (Wilcoxon signed rank test, *p *= 0.66, Figure 4).

## Discussion

The purpose of this study was to evaluate the potential toxicity of ^18^F-FLT when administered in a radiotracer dose for PET imaging. Such a study was made necessary because of previously reported toxicity in clinical trials employing therapeutic doses. In 1994, Flexner et al. published a concentration-control trial involving initially 14 asymptomatic HIV-positive patients treated with FLT over a period of 16 weeks. These patients received a de-escalating oral dose of FLT starting from 0.125 mg/Kg (AUC_12 _~ 417 ng*h/mL) every 12 hours to reach a target area under the curve of 300 ng*h/mL for a 12 hour dosing interval [17]. Serious grade III hematologic toxicity (anemia and leukopenia) was observed with concentrations greater than 300 ng*h/mL. Grade II+ anemia was observed within 4 weeks with a concentration of 300 ng*h/mL. Subsequently, a randomized, double-blind phase I/II trial was designed to characterize FLT pharmacokinetics, anti-viral activity, and toxicity. Forty-eight patients were randomized into 3 groups with different target areas of FLT concentration (50 ng*h/mL, 100 ng*h/mL, or 200 ng*h/mL) for up to 16 weeks of treatment. Grade II+ anemia developed at doses greater than 100 ng*h/mL, granulocytopenia at doses greater than 200 ng*h/mL, and mild peripheral neuropathy within 40 days at any dose. The bone marrow toxicity was reversible when the drug was discontinued. This type of toxicity was not seen with reduced FLT doses. However, this phase I/II trial was suspended because two patients died from acute hepatic failure after 12 weeks of therapy, one in Flexner's study at AUC_12 _of 200 ng*h/mL, the other from a European trial using a 10 mg/day regimen. Therefore, the FLT toxicology data reported for human therapeutic use shows the lowest toxicity regimen tested to be 50 ng*h/mL for a 12 hour dosing interval which led to only mild peripheral neuropathy within a mean of 40 days of therapy [17]. Therefore, in addition to discussing our toxicology data for a single radiotracer dose of ^18^F-FLT, we will compare this dose level to the multiple larger doses used in this lowest toxicity clinical regimen.

We evaluated the toxicity of ^18^F-FLT in a series of 20 patients who underwent ^18^F-FLT PET imaging. We took two different approaches to this evaluation: first, a direct laboratory measure of electrolytes, hepatic, metabolic, and renal function of these patients; second, a comparison of the maximal amount of FLT compound injected for a ^18^F-FLT PET study to the therapeutic dose shown to be the least toxic in clinical trials. To this end, AUC_12 _values for our study patients were calculated from both imaging-derived TACs and actual blood samples.

No change was found in the renal function (BUN and creatinine levels) of the 20 patients studied or in the following hepatic function measures: AST, ALT, alkaline phosphatase, and total bilirubin. As described in the results section, decreases in the patients' average hematocrit, RBC and hemoglobin levels were observed. One of the challenges of this study is that it was designed as a companion analysis to a primary imaging study performed in potentially resectable NSCLC. As a result, seventeen of 20 (85%) patients studied underwent surgical staging (mediastinoscopy and/or video assisted thoracoscopy) with 14/20 also undergoing a tumor resection procedure, at the same time as the mediastinoscopy, under general anaesthesia after ^18^F-FLT PET. Thirteen of the 14 (93%) had resection within 1 week of the ^18^F-FLT PET study, 7 of these were the day after the ^18^F-FLT PET study. Because surgery involves blood loss and metabolic stress, hematological values were consequently affected and a long-term relationship between the ^18^F-FLT PET study and hematological values is difficult to establish. Nonetheless, when we only consider pre-surgical lab values and all lab values for patients who did not undergo any surgical procedure, analysis of the data reveals a single small early drop in hemoglobin, hematocrit and RBC. This suggests that the additional drop observed in these parameters at later time points for the whole population is due to surgical blood loss. The initial decrease in hemoglobin, hematocrit and RBC is probably attributable to a dilution effect since patients were hydrated with 500 cc of IV normal saline during the PET scan. Giving more credibility to the dilution hypothesis, haptoglobin measurements performed on the last 3 patients having demonstrated such a RBC decrease showed no evidence of hemolysis at the end of the ^18^F-FLT PET imaging session. Platelet and white blood cell counts did not show any statistically significant differences over time and no immediate effect of dilution. This is likely because immediately available pools of such cells exist in the body, helping to maintain their levels despite hydration. Another important point is that the patients whose medical records contain laboratory values for times late after ^18^F-FLT PET are those that had a more prolonged post-operative course with more severe alterations expected in their laboratory values.

Statistical testing showed that the patients' albumin level decreased slightly over time but its level remained within normal limits. However, Bonferroni post-test analysis shows that this change arises from the initial decrease (11.5%) between the pre-^18^F-FLT PET value and the immediate post-^18^F-FLT PET blood draw. This decrease is also likely explained by the hemodilution.

No change was observed in the results of the neurological examinations performed before and immediately after the ^18^F-FLT PET study. Furthermore, a review of the clinical records for the 4 months following ^18^F-FLT imaging revealed no new neurological complaints.

The second approach used to estimate the toxicity of a radiotracer dose of ^18^F-FLT is based on a comparison of the corresponding AUC_12 _to the AUC_12 _of the lowest and least toxic therapeutic regimen (50 ng*h/mL). For a 5 mCi radiotracer dose of ^18^F-FLT and the lowest accepted specific activity of 0.1 Ci/μmol, the imaging-derived AUC_12 _values ranged from 0.405 to 1.262 ng*h/mL with a mean of 0.770 ± 0.285 ng*h/mL. This corresponds to 0.81% to 2.52% of the lowest and least toxic clinical trial AUC_12 _of 50 ng*h/mL. The blood samples-derived AUC_12 _values ranged from 0.232 to 1.34 ng*h/mL with a mean of 0.802 ± 0.303 ng*h/mL. This corresponds to 0.46% to 2.68% of the least toxic clinical trial. Therefore, the AUC_12 _of a single 5 mCi radiotracer dose of ^18^F-FLT is very small compared to the AUC_12 _of the least toxic therapeutic trial. In addition, one should realize that the latter trial administered FLT every 12 hours for 112 days with the only toxicity, peripheral neuropathy, developing at a mean of 40 days. A conservative comparison of a radiotracer dose of ^18^F-FLT to 80 therapeutic doses of FLT shows that 5 mCi of FLT represents at most 2.68%/80 or nearly 3,000 times less FLT administered than in Flexner's least toxic regimen, a considerable difference. Therefore, this provides further justification of the expected negligible toxicity from 5 mCi of ^18^F-FLT for PET imaging. Alterations in white blood cell count reported by Flexner et al at higher AUC_12 _regimens were not observed in our patients. Furthermore, by radiochemistry requirements, the lowest acceptable specific activity for ^18^F-FLT at the end of synthesis is 0.1 Ci/μmol. For a maximal injected dose of 5 mCi, this would correspond to a maximum unlabeled drug dose of 12.2 μg of FLT. Using the correspondence between a 0.125 mg/Kg dose and an AUC_12 _of 417 ng*hr/mL reported by Flexner [17], the least toxic dose of 50 ng*hr/mL corresponds to a dose of 0.015 mg/Kg, or 1.05 mg every 12 hours for a 70 Kg person. Over 40 days (80 doses), this is 1.05 mg × 80 doses or a 6,885-fold greater dose than that involved in a radiotracer administration of [^18^F]FLT. In practice, the typical specific activity levels were 3 to 4 times higher than the acceptable level of 0.1 Ci/μmol. This implies that for a 5 mCi ^18^F-FLT dose, even smaller amounts of FLT are injected.

## Conclusion

This study shows no evidence of toxicity or complication attributable to a 0.07 mCi/Kg (max 5 mCi) dose of ^18^F-FLT intravenously injected in the 20 patients studied. In addition, we provided careful estimation of the relative amount of FLT involved in a 5 mCi radiotracer dose for comparison to that administered in previously reported clinical trials. We believe that the use of such a radiotracer dose of ^18^F-FLT for PET imaging in human subjects is safe. Since ^18^F-FLT dosimetry is also known [15], widespread clinical PET imaging with ^18^F-FLT can now be undertaken.

## List of abbreviations

^18^F-FLT: 3'-deoxy-3'- [^18^F]fluorothymidine

AUC_12_: area under the curve for 12 hours

BUN: blood urea nitrogen

NSCLC: non-small cell lung cancer

PET: positron emission tomography

RBC: red blood cell count

TAC: time-activity curve

TK-1: thymidine kinase-1

WBC: white blood cell count

## Competing interests

The author(s) declare that they have no competing interests.

## Authors' contributions

ET collated and analyzed the data, and wrote significant portions of the manuscript. LW coordinated the study, compiled the patient information and study results, and helped write the paper. JG formulated the ^18^F-FLT doses and provided input on the process and the properties of the drug. LP counted the radioactivity in blood samples and provided input on generating the TACs and AUC_12 _values. MW oversaw the analysis of the laboratory studies and provided input on interpreting the results. HV conceptualized the study, obtained institutional approval, oversaw the acquisition of subjects and data, and wrote and edited the paper with ET.

## Pre-publication history

The pre-publication history for this paper can be accessed here:


